# Manipulation of the Gut Microbiota Reveals Role in Colon Tumorigenesis

**DOI:** 10.1128/mSphere.00001-15

**Published:** 2015-11-04

**Authors:** Joseph P. Zackular, Nielson T. Baxter, Grace Y. Chen, Patrick D. Schloss

**Affiliations:** a Department of Microbiology and Immunology, University of Michigan, Ann Arbor, Michigan, USA; b Department of Internal Medicine, Division of Hematology and Oncology, University of Michigan, Ann Arbor, Michigan, USA; Joint Genome Institute, Lawrence Berkeley National Laboratory

**Keywords:** 16S rRNA gene sequencing, azoxymethane, colorectal cancer, dextran sodium sulfate, microbial ecology, microbiome, murine models

## Abstract

There is growing evidence that individuals with colonic adenomas and carcinomas harbor a distinct microbiota. Alterations to the gut microbiota may allow the outgrowth of bacterial populations that induce genomic mutations or exacerbate tumor-promoting inflammation. In addition, it is likely that the loss of key bacterial populations may result in the loss of protective functions that are normally provided by the microbiota. We explored the role of the gut microbiota in colon tumorigenesis by using an inflammation-based murine model. We observed that perturbing the microbiota with different combinations of antibiotics reduced the number of tumors at the end of the model. Using the random forest machine learning algorithm, we successfully modeled the number of tumors that developed over the course of the model on the basis of the initial composition of the microbiota. The timing of antibiotic treatment was an important determinant of tumor outcome, as colon tumorigenesis was arrested by the use of antibiotics during the early inflammation period of the murine model. Together, these results indicate that it is possible to predict colon tumorigenesis on the basis of the composition of the microbiota and that altering the gut microbiota can alter the course of tumorigenesis.

**IMPORTANCE** Mounting evidence indicates that alterations to the gut microbiota, the complex community of bacteria that inhabits the gastrointestinal tract, are strongly associated with the development of colorectal cancer. We used antibiotic perturbations to a murine model of inflammation-driven colon cancer to generate eight starting communities that resulted in various severities of tumorigenesis. Furthermore, we were able to quantitatively predict the final number of tumors on the basis of the initial composition of the gut microbiota. These results further bolster the evidence that the gut microbiota is involved in mediating the development of colorectal cancer. As a final proof of principle, we showed that perturbing the gut microbiota in the midst of tumorigenesis could halt the formation of additional tumors. Together, alteration of the gut microbiota may be a useful therapeutic approach to preventing and altering the trajectory of colorectal cancer.

## Abstract (precis)

Mounting evidence indicates that alterations to the gut microbiota, the complex community of bacteria that inhabits the gastrointestinal tract, are strongly associated with the development of colorectal cancer. We used antibiotic perturbations to a murine model of inflammation-driven colon cancer to generate eight starting communities that resulted in various severities of tumorigenesis. Furthermore, we were able to quantitatively predict the final number of tumors on the basis of the initial composition of the gut microbiota. These results further bolster the evidence that the gut microbiota is involved in mediating the development of colorectal cancer. As a final proof of principle, we showed that perturbing the gut microbiota in the midst of tumorigenesis could halt the formation of additional tumors. Together, alteration of the gut microbiota may be a useful therapeutic approach to preventing and altering the trajectory of colorectal cancer.

## INTRODUCTION

The mammalian gastrointestinal tract is home to a complex and dynamic community of microorganisms, termed the gut microbiota, that is essential for maintaining host health (1). There are complex interactions among bacterial populations in the gut that have an important effect on host health (2–4). The number of diseases that are associated with abnormalities in the gut microbiota highlights the importance of these ecological interactions (5–7). Over the last several years, it has been well documented that perturbations to this community are associated with colorectal cancer (CRC) in humans and mice (8–15). We have previously shown that CRC-associated changes in the gut microbiota directly potentiate colon tumorigenesis in a mouse model of CRC (16). In that study, we observed clear shifts in the microbiota that were associated with a stepwise progression in the number of tumors that developed in the colon. In addition, we showed that transfer of the tumor-associated microbiota to germfree mice resulted in increased tumor formation relative to that in germfree mice that received the microbiota of healthy mice. These results were supported by a subsequent study in which we colonized germfree mice with the microbiota of human donors and observed that different starting communities yielded significant variation in the number of tumors that formed (17). Combined, these results demonstrate that the microbiota interacts with the host to affect tumor susceptibility. A critical question that remains unanswered is what factors and ecological principles mediate the gut microbiota’s influence on tumor development. Deciphering how changes in microbial community composition and structure alter gut homeostasis and subsequently modulate tumorigenesis is an essential step in understanding the etiology of CRC.

Several bacterial populations, including *Escherichia coli*, *Bacteroides fragilis*, and *Fusobacterium nucleatum*, have been shown to directly influence tumor development in the colon. The mechanisms by which bacteria potentiate these processes range from the production of carcinogenic toxins (18, 19) to direct manipulation of the inflammatory status of the tumor microenvironment (20, 21). Although individual bacterial populations undoubtedly modulate colorectal carcinogenesis, there are likely a myriad of commensal bacteria that work together to influence tumorigenesis in the colon. This is supported by several studies that have explored the gut microbiota associated with individuals with CRC (8–15, 22). With each study, the number of CRC-associated bacterial populations that likely play a role in tumorigenesis continues to grow. This is likely due to the fact that there is significant functional redundancy within the gut microbiota and various bacterial populations may fill similar roles in tumorigenesis (23–25). Furthermore, some bacterial populations have been hypothesized to be protective against CRC (26, 27). This protective phenotype may be mediated through metabolite production, induction of immunotolerance, or an ability to outcompete pathogenic bacteria (28). We hypothesize that multiple bacteria in the gut microbiota have the potential to play protumorigenic or tumor-suppressive roles; thus, the gut microbiota’s influence on CRC is likely to be driven by complex interactions within the microbiota and the colonic epithelium.

We have shown that conventionally raised mice treated with a cocktail of metronidazole, streptomycin, and vancomycin in their drinking water had a significant decrease in tumor numbers by using an inflammation-based model of CRC (22). In the present study, we explored how differential alterations in the microbiota by different antibiotic treatments affected the composition of the microbiota and how changes in bacterial community structure affected tumor susceptibility. Our results confirmed our hypothesis that the microbiota is capable of driving tumorigenesis and that an antibiotic-based intervention during tumor induction can arrest tumorigenesis. Our analysis further supports a model in which individual bacterial populations play an important role in CRC, but the ecological interactions and community structure of the gut microbiota mediate the capacity to modulate tumorigenesis.

## RESULTS

### Antibiotic perturbation of the gut microbiota modulates tumorigenicity.

**FIG 1:**
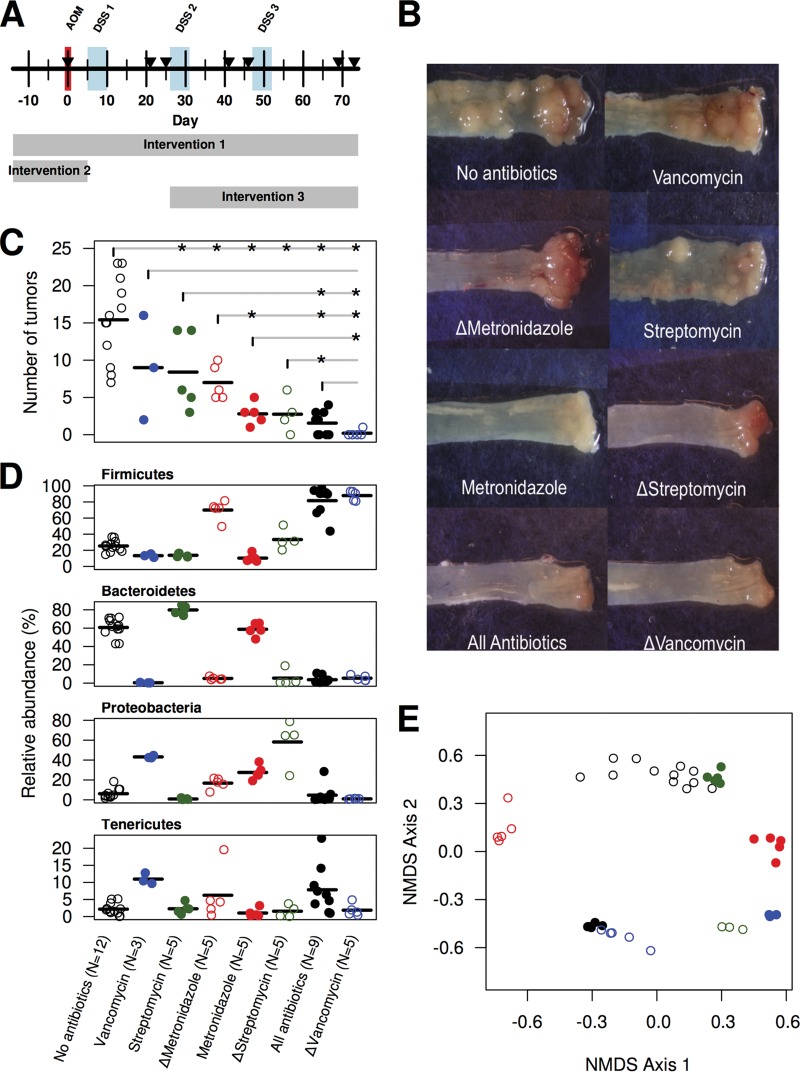
Antibiotic perturbation drives changes in microbial community structure and the final tumor burden. The AOM-DSS model was administered to C57BL/6 mice reared under SPF conditions with different antibiotic perturbations that were applied during the period covered by each of the rectangles; The arrowheads indicate the times when fecal samples that were used for our analysis were obtained (A). The mice were treated with all of the possible combinations of metronidazole, streptomycin, and vancomycin to create eight treatment groups, which resulted in a continuum of tumor burdens in the mice (B to D). The stars indicate which treatments yielded a significantly (*P* < 0.05) different number of tumors compared to the treatment with the vertical line. The antibiotic treatments resulted in variation in the taxonomic structure of the communities at the start of the model (day 0) (D). The two-dimensional nonmetric multidimensional scaling ordination had a stress of 0.20 and explained 84.0% of the variation in the distances (E).

We subjected specific-pathogen-free (SPF) C57BL/6 mice to an inflammation-based model of CRC that utilizes azoxymethane (AOM) as a mutagen and dextran sodium sulfate (DSS) to induce inflammation (16, 17, 29) (Fig. 1A). To determine how differential changes in the gut microbiota affect tumorigenesis, we manipulated the microbiota by administering seven different antibiotic combinations for the length of the model (intervention 1; Fig. 1A) and then quantified the effects of the treatments on the number of tumors observed at the end of the model (Fig. 1B and C). Specifically, we treated mice with (i) no antibiotics; (ii) metronidazole, streptomycin, and vancomycin (all of the antibiotics); (iii) streptomycin and vancomycin (Δmetronidazole); (iv) metronidazole and vancomycin (Δstreptomycin); (v) metronidazole and streptomycin (Δvancomycin); (vi) metronidazole; (vii) streptomycin; or (viii) vancomycin. The three antibiotics were selected for their reported ability to target general groups of bacteria, including anaerobes (metronidazole), Gram-negative organisms (streptomycin), and Gram-positive organisms (vancomycin). Upon necropsy, we observed that perturbation of the microbiota through the use of antibiotics yielded a differential capacity for colon tumorigenesis (Fig. 1B and C). Sequencing of the 16S rRNA genes that were present in the feces of conventional and antibiotic-treated mice demonstrated that the different antibiotic treatments generated different bacterial communities prior to AOM injection (Fig. 1D); however, the composition of these communities could not have been predicted by the spectrum of the antibiotic that was used to treat the mice. The eight community structures generated by using the untreated mice and those that received one of the seven antibiotic combinations were all significantly different from each other (all *P* < 0.05 by analysis of molecular variance with Benjamini-Hochberg correction). These results indicated that the communities are distinct from each other (Fig. 1E) and varied in the ability to drive tumorigenesis.

### Tumor burden can be predicted from the initial microbiota.

**FIG 2:**
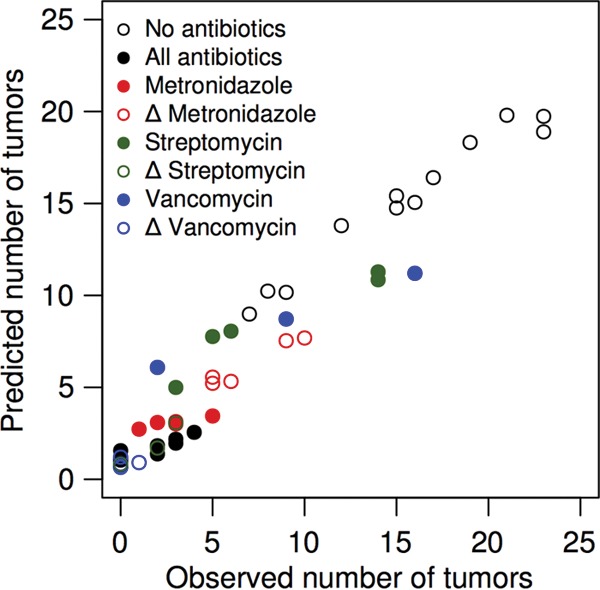
A random forest model successfully predicted the number of tumors in the mice at the end of the model on the basis of their microbiota composition at the start of the model. The model included 12 OTUs and explained 67.7% of the variation in the data.

**FIG 3:**
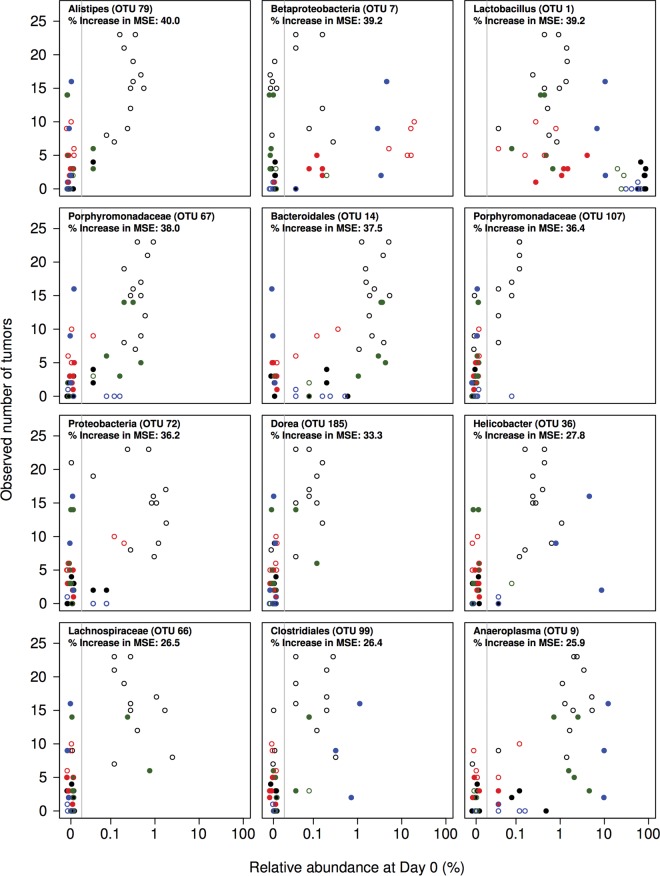
Relationship between the initial relative abundance of the most informative OTUs from the random forest model and the number of tumors found in the mice at the end of the model. The vertical gray line indicates the limit of detection. Panels are ordered in decreasing order of the percent increase in the mean square error (MSE) of the model when that OTU was removed. The color and shape of the plotting symbols correspond to those used in Fig. 2.

Serial collection of fecal samples allowed us to ascertain the composition of the microbiota for each mouse and associate it with the number of tumors that developed at the end of the model. Using the 16S rRNA gene sequence data generated from feces collected on the day of AOM injection, we assigned the sequences to operational taxonomic units (OTUs) that were defined as a group of sequences that, on average, were not more than 3% different from each other. We then used the regression-based random forest machine learning algorithm to identify OTUs that would enable us to predict the number of tumors that developed at the end of the model. The model that included all 685 OTUs explained 53.9% of the variation in the tumor counts. We then sorted the OTUs by their importance in the random forest model as determined by the percent reduction in the mean square error when that OTU was removed from the model. There was a peak in the amount of the variation explained in the observed tumor counts when we used the 12 most important OTUs (see Fig. S1 in the supplemental material). The simplified model with 12 OTUs explained 67.7% of the variation in the observed tumor counts (Fig. 2). These 12 OTUs included members of the phyla *Firmicutes* (OTUs 1, 66, 99, and 185), *Bacteroidetes* (OTUs 14, 67, 79, and 107), *Proteobacteria* (OTUs 7, 36, and 72), and *Tenericutes* (OTU 9). With the exception of OTUs affiliated with members of the genus *Lactobacillus* (OTU 1) and the class *Betaproteobacteria* (OTU 7), each of the OTUs was associated with an increased tumor burden (Fig. 3). The relative abundance of the *Lactobacillus*-affiliated OTU at the start of the model was inversely proportional to the tumor burden at the end of the model. There was not a clear relationship between the initial relative abundance of the *Betaproteobacteria*-affiliated OTU and the final tumor burden. Interestingly, tumorigenesis was not exclusively dependent on the presence of any of the OTUs. In other words, tumors could form even when any specific tumor-predictive OTU was below the limit of detection. This suggests that the role of the microbiota in driving tumor formation was context dependent. More broadly, the random forest model demonstrated that it was possible to predict the number of tumors at the end of the model on the basis of the composition of the microbiota at the beginning of the model.

10.1128/mSphere.00001-15.1 Figure S1  Effect of pruning the number of OTUs included in the random forest model for predicting the number of tumors at the end of the model on the basis of the microbiota found at the start of the model. The order of OTUs was set by the percent increase in mean square error when that OTU was removed from the model. The percentage of the variance explained here indicates the quality of the fit when the top features were used to generate a model. The star indicates the number of OTUs that resulted in the model explaining the maximum percentage of the variance. Download Figure S1, PDF file, 0.04 MB. Copyright © 2015 Zackular et al. 2015 Zackular et al. This content is distributed under the terms of the Creative Commons Attribution 4.0 International license.

### Tumor burden can be predicted from the microbiota at the end of the model.

Similar to our analysis using the initial composition of the microbiota, we developed a random forest regression model to predict the number of tumors in the mice on the basis of the composition of the microbiota at the end of the model (see Fig. S2 in the supplemental material). The simplified model included eight OTUs and explained 65.6% of the variation in the tumor counts (see Fig. S3). This was comparable to what we observed when we modeled tumor counts on the basis of the initial community composition. This model utilized the relative abundance data from OTUs affiliated with members of the phyla *Firmicutes* (OTU 85), *Bacteroidetes* (OTUs 7, 19, 28, 29, and 51), *Proteobacteria* (OTU 7), and “*Candidatus* Saccharibacteria” (OTU 192; see Fig. S4). Interestingly, of the OTUs that were predictive of the tumor counts based on the initial and final community composition data, only one of the OTUs overlapped, which was affiliated with the class *Betaproteobacteria* (OTU 7). The distinction between OTUs that were predictive of tumor burdens on the basis of the community compositions at the beginning and end of the model suggests that the communities that gave rise to tumors were different from those that were enriched in a tumor-laden environment.

10.1128/mSphere.00001-15.2 Figure S2  Effect of pruning the number of OTUs included in the random forest model for predicting the number of tumors at the end of the model on the basis of the microbiota found at the end of the model. The order of OTUs was set by the percent increase in mean square error when that OTU was removed from the model. The percentage of the variance explained here indicates the quality of the fit when the top features were used to generate a model. The star indicates the number of OTUs that resulted in the model explaining the maximum percentage of the variance. Download Figure S2, PDF file, 0.04 MB. Copyright © 2015 Zackular et al. 2015 Zackular et al. This content is distributed under the terms of the Creative Commons Attribution 4.0 International license.

10.1128/mSphere.00001-15.3 Figure S3  A random forest model successfully predicted the number of tumors in the mice at the end of the model on the basis of their microbiota composition at the end of the model. The model included eight OTUs and explained 65.6% of the variation in the data. Download Figure S3, PDF file, 0.04 MB. Copyright © 2015 Zackular et al. 2015 Zackular et al. This content is distributed under the terms of the Creative Commons Attribution 4.0 International license.

10.1128/mSphere.00001-15.4 Figure S4  Relationship between the final relative abundance of the most informative OTUs from the random forest model and the number of tumors found in the mice at the end of the model. The vertical gray line indicates the limit of detection. Panels are ordered in decreasing order of the percent increase in the mean square error of the model when that OTU was removed. The color and shape of the plotting symbols correspond to those used in Fig. S3. Download Figure S4, PDF file, 0.1 MB. Copyright © 2015 Zackular et al. 2015 Zackular et al. This content is distributed under the terms of the Creative Commons Attribution 4.0 International license.

### The microbial community is dynamic during inflammation-associated tumorigenesis.

**FIG 4:**
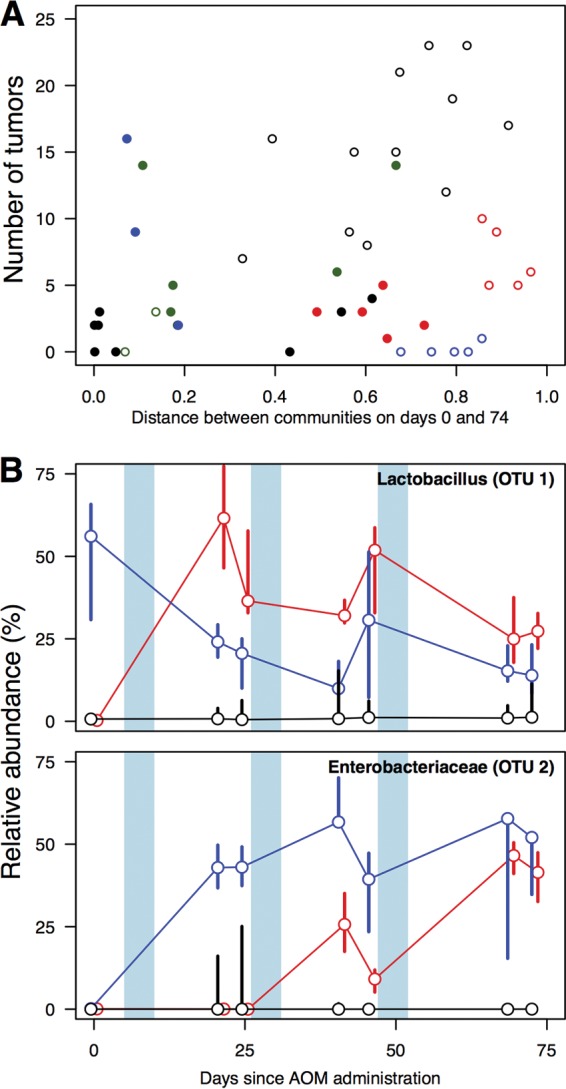
The murine microbiota is dynamic, but the amount of change is not associated with the final number of tumors. The structure of the gut microbiota of the untreated, Δmetronidazole-treated (open red circles), and Δvancomycin-treated (open blue circles) mice changed the most throughout the model as measured with the θ_YC_ distance metric (A). OTUs 1 and 2 were among the most dynamic OTUs across all of the treatment groups; here we depict the change in their relative abundance across the model in those treatment groups that experienced the greatest overall change in community structure (B). The plotting symbols and characters are the same as those used in Fig. 1. In panel B, the median relative abundance is indicated by the plotting symbol and the range of observed relative abundances is plotted by the vertical bar. The vertical blue regions indicate when the DSS treatments were applied.

Using mice that were colonized with human feces, we previously reported that tumor burden was associated with the amount of change in the community structure over the course of the AOM-DSS model (17). In the present study, however, there was a nonsignificant association between the change in the community structure as measured by the θ_YC_ metric of community structure similarity and tumor burden (ρ = 0.26, *P* = 0.08; Fig. 4A). We did observe that mice that did not receive antibiotics and those that received the Δvancomycin and Δmetronidazole treatments changed the most over the course of the model. When we identified those OTUs whose relative abundances changed the most across each treatment group, we found that OTUs affiliated with the genus *Lactobacillus* (OTU 1) and the family *Enterobacteriaceae* (OTU 2) were consistently among the most dynamic OTUs across the treatment groups (Fig. 4B). Interestingly, the initial relative abundance of the *Lactobacillus*-affiliated OTU was predictive of the final tumor burden, but the final relative abundance of neither OTU was predictive of the final tumor burden. These data suggest that the magnitude of the change that occurs across a microbial community during tumorigenesis is not strongly associated with the tumor burden. Instead, the relative abundance of a subset of populations within the community dictates the tumor burden.

### Antibiotic intervention during inflammation reduces tumorigenesis.

**FIG 5:**
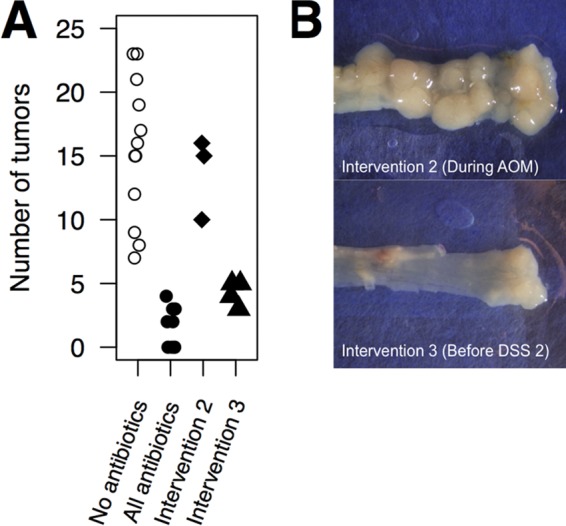
Antibiotic intervention prior to a second administration of DSS alleviates the tumor burden. Interventions with an antibiotic cocktail of metronidazole, vancomycin, and streptomycin were performed as depicted in Fig. 1A, and enumeration of tumors was performed at the endpoint of the model (A). Representative images of tumors in the distal colons of mice from each treatment group (B).

The results of our present study and our previous investigations of the role of the gut microbiota in colonic tumorigenesis have suggested that by manipulating the gut microbiota, it would be possible to manipulate tumorigenesis (16, 17). To further validate these results, we performed two additional antibiotic intervention experiments. We first treated mice with vancomycin, metronidazole, and streptomycin 2 weeks prior to the administration of AOM and up until the first round of DSS and then removed the antibiotic cocktail for the remainder of the model (intervention 2; Fig. 1A). We found that these mice had a tumor burden similar to that of untreated mice (Fig. 5). Next, we treated mice after the first round of DSS administration with the antibiotic cocktail until the end of the model. Our previous work found that the period following the first round of DSS coincided with a period when inflammatory responses were the greatest and there were aberrant changes in the gut microbiota (16) (intervention 3; Fig. 1A). With these mice, we found that the intervention resulted in a significant decrease in the number of tumors (Fig. 5). These results suggest that the gut microbiota-mediated effect on CRC is independent of AOM-mediated carcinogenesis. Furthermore, it shows that targeting of the gut microbiota at later stages of tumor growth is a viable option for minimizing tumorigenesis and highlights microbiota manipulation as a potential therapeutic in CRC.

## DISCUSSION

In the present study, we established the importance of the microbial community structure in determining the extent of tumorigenesis. We demonstrated that manipulation of the murine gut microbiota with different antibiotic cocktails resulted in distinct community structures that were associated with disparate levels of tumorigenesis. To determine whether the microbiota was involved in possibly converting the AOM to a carcinogenic metabolite or involved in the inflammation process, we restricted the application of antibiotics to alter the microbiota during these phases of the model. We determined that the gut microbiota affects tumorigenesis via a mechanism that does not involve AOM-induced carcinogenesis. Our experiments also demonstrated that targeting of the gut microbiota at the emergence of dysbiosis (i.e., after the first round of DSS in the AOM/DSS model) is a viable strategy for the amelioration of colon tumorigenesis. Such a result offers hope that by altering a person’s gut microbiota, it may be possible to alter that person’s risk of developing colon cancer.

Our analysis suggests that community-wide changes affect the process of tumorigenesis in the murine gut. To investigate this process, we manipulated the gut microbiota by applying various antibiotic cocktails. One risk of this approach is that the antibiotic perturbation could reduce the overall bacterial load and confound the analysis. We previously analyzed the feces of mice receiving all three antibiotics by using a culture-independent quantitative PCR approach and observed a nonsignificant reduction in the bacterial load (22). This result agrees with other studies that have used similar antibiotic cocktails to study the role of the microbiota in colitis (30, 31). Meanwhile, others have seen a small but significant decrease in the bacterial load that varied along the gastrointestinal tract (32). Considering our previous result and the fact that we observed a relatively consistent relationship between bacterial populations in the gut and tumor burden, it is unlikely that differences in the bacterial load of the colon are responsible for the results observed. An alternative approach involves colonizing germfree mice with defined cocktails of bacteria or from murine or human donors. The challenge of this approach is that the immune system would still be altered from a normal state, and it is difficult to dictate the final structure of a transplanted community (17). By pursuing various approaches to generate variation in the initial community, it is clear that the gut microbiota is involved in protecting against and exacerbating colonic tumorigenesis.

There has been a focus on identifying specific bacterial populations that are etiologic agents of CRC. Several commensal bacteria, including *E. coli*, *F. nucleatum*, and enterotoxigenic *B. fragilis* (ETBF) have been linked to CRC in humans (18, 19, 21). *F. nucleatum*, which has been detected on the surface of over 50% of adenomas, can promote inflammation within the tumor microenvironment in multiple intestinal neoplasia mice (10, 20). ETBF increases tumor multiplicity in the colons of multiple intestinal neoplasia mice through the action of a secreted metalloprotease toxin. It has been estimated that between 5 and 35% of people carry ETBF (33). Although there is substantial evidence of a role in the potentiation of tumorigenesis, the fact that each of these bacteria is associated with only a fraction of CRCs suggests that it is unlikely that there is a single microbial agent that causes cancer. Rather, the role of the gut microbiota in CRC is likely polymicrobial in nature. Our results support this hypothesis, as we demonstrated that different community structures were associated with similar levels of tumorigenesis in mice. When we examined the relative abundance of bacterial populations associated with an increased tumor burden, we never observed consistent enrichment of any one population across all of the treatment groups (Fig. 3). Similar to a previous study exploring the role of the gut microbiota in shaping resistance to *Clostridium difficile* colonization (34), we found that the context of the gut microbiota is important in predicting the eventual tumor burden. Such a result suggests that there may be redundancy in tumor-modulating roles among different bacterial populations within the gut microbiota.

As described above, there has been a considerable effort to identify bacteria and their products that cause colon cancer. In contrast, our results indicate a need to focus on protective populations. We consistently observed that the relative abundance of a *Lactobacillus*-affiliated OTU (OTU 1) was predictive of a light tumor burden (Fig. 3). Various *Lactobacillus* strains are widely used as probiotics to reduce inflammation in the gastrointestinal tract. These bacteria have been shown to reduce inflammation in mouse models of colitis (35), necrotizing enterocolitis (36), and graft-versus-host disease (37). *Lactobacillus* spp. enhance epithelial barrier function by inducing the production of mucus and tight-junction proteins (38, 39) and can modulate the host’s immune response by suppressing the expression of the proinflammatory cytokine interleukin-17 (40). The clinical significance of this result is unclear, however, considering that we observed suppression of tumorigenesis when the microbiota had levels of *Lactobacillus* bacteria that were higher than the 0.1 to 1% relative abundance commonly observed in the feces of humans (41). Regardless, a better understanding of the possible protective role of *Lactobacillus* bacteria in limiting tumorigenesis may be useful in developing probiotic and prebiotic therapies.

It is striking that we were able to quantitatively predict the tumor burden that resulted at the end of our 73-day model on the basis of the community composition at the start of the model. The random forest regression modeling approach is nonparametric and accounts for the nonlinearities and interactions within the data set to identify a subset of OTUs that are predictive of the tumor burden. An added advantage of this approach is that cross-validation is built into the model generation procedure, limiting the risks of overfitting the model to the data (42). The regression-based approach has been used with microbiome data to predict *C. difficile* colonization (34) and to assign a microbiome-based age to malnourished children (43). Given the significant heterogeneity that we observe in the gut microbiota, regression-based random forest models are a powerful tool to identify subsets of communities that are associated with disease.

Dysbiosis of the gut microbiota generates a proinflammatory environment that results in a self-reinforcing pathogenic cascade between the gut microbiota and the host (16, 17). In this study, we demonstrated that antibiotic manipulation of the gut microbiota during the onset of inflammation can significantly decrease tumorigenesis in mice. This highlights the efficacy of targeting the gut microbiota in CRC. Additional studies are needed to explore the viability of manipulating the gut microbiota in CRC by methods such as diet, probiotics, and prebiotics.

## MATERIALS AND METHODS

### Animals and animal care.

Studies were conducted with adult (8 to 12 weeks old) age-matched C57BL/6 male mice that were maintained under SPF conditions. Mice were cohoused in groups of five and fed the same autoclaved chow diet. All animal experiments were approved by the University Committee on Use and Care of Animals at the University of Michigan and carried out in accordance with the approved guidelines.

### Inflammation-induced colon tumorigenesis.

Mice received a single intraperitoneal injection of AOM (10 mg/kg). Water containing 2% DSS was administered to mice beginning on day 5 for 5 days; this followed by 16 days of plain water. This was repeated twice for a total of three rounds of DSS (16). Mice were euthanized 3 weeks after the third round of DSS administration for tumor counting. At necropsy, all colons were harvested, flushed of luminal contents, and cut open longitudinally to count and measure tumors.

### Antibiotic treatment.

Mice were treated with all of the possible combinations of metronidazole (0.75 g/liter), streptomycin (2 g/liter), and vancomycin (0.5 g/liter) to create the following eight treatment groups: no antibiotics (*n =* 12), all of the antibiotics (metronidazole, streptomycin, and vancomycin; *n* = 9), Δmetronidazole (streptomycin and vancomycin; *n* = 5), Δstreptomycin (metronidazole and vancomycin; *n* = 5), Δvancomycin (metronidazole and streptomycin; *n* = 5), metronidazole only (*n =* 5), streptomycin only (*n =* 5), and vancomycin only (*n =* 3). Antibiotics were administered in mouse drinking water for 2 weeks prior to and throughout the duration of AOM/DSS administration, unless otherwise specified in Fig. 1A. Tumors were enumerated at the end of the model.

### DNA extraction and 16S rRNA gene sequencing.

Fecal samples were collected daily from the mice throughout the AOM/DSS protocol and immediately frozen for storage at −20°C. For each mouse, eight fecal samples distributed over the 73-day timeline of the AOM/DSS model were selected for analysis (Fig. 1A). Microbial genomic DNA was extracted with the PowerSoil-htp 96 Well Soil DNA Isolation kit (Mo Bio Laboratories) with an EpMotion 5075. The V4 region of the 16S rRNA gene from each sample was amplified, sequenced with the Illumina MiSeq Personal Sequencing platform, and curated with the mothur software package as described previously (44, 45). Briefly, we reduced sequencing and PCR errors by requiring reads to fully overlap, and in cases where base calls conflicted, we broke the conflict by requiring one base call to have a Phred quality score 6 units higher than the other; otherwise, the base call was replaced with an ambiguous base call in the contig. Any reads containing ambiguous base calls were culled. Sequences were aligned to a customized version of the SILVA 16S rRNA sequence database (46) and were screened to ensure that they correctly overlapped within the V4 region. The resulting sequences had a median length of 253 nucleotides. Chimeric sequences were identified with the *de novo* implementation of UCHIME, and they were culled (47). A mock community was sequenced and processed in parallel with the fecal samples. On the basis of the mock community data, we observed a sequencing error rate of 0.05%. Cleaned sequences were assigned to OTUs by using the average neighbor clustering algorithm such that the sequences within an OTU, on average, were not more than 3% different from each other (48). We obtained a majority consensus classification for each OTU by using the classification of each sequence obtained with a naive Bayesian classifier trained against a training set from the Ribosomal Database Project (version 10) as implemented in mothur; we required a minimum confidence score of 80% (49, 50). Distances between communities were calculated with the θ_YC_ distance metric, which incorporates the overlap in the membership and abundance of OTUs between pairs of communities (51). To limit effects of uneven sampling, we rarefied each sample to 2,500 sequences per sample before calculating the θ_YC_ distance; our analysis used the average distance matrix based on 100 randomizations.

### Statistical analysis.

The microbiota data were analyzed with the R Project for Statistical Computing. Our modeling analysis utilized the regression-based random forest machine learning algorithm, which utilizes a decision tree-based approach that accounts for nonlinear data and interactions among features and includes an internal cross-validation to prevent overfitting. For each tree, two-thirds of the samples were randomly selected to train the model and one-third of the samples were selected to test the model. All random forest models were made by using the randomForest package with 10,000 trees (42). Random forest regression models were constructed with the OTU count data obtained with one random subsampling of 2,500 sequences per sample. The models were trained to predict the number of tumors observed at the end of the model. Diagnostic plots indicated that the percentage of the variance explained had stabilized with this number of trees. Comparisons of tumor counts were made by carrying out nonparametric pairwise Wilcoxon tests. The resulting *P* values were corrected for multiple comparisons with the Benjamini-Hochberg procedure by using an experiment-wide type I error rate of 0.05.

### Availability of code and sequencing data.

The complete analysis methods used and this document as an R-executable document are available at https://github.com/SchlossLab/Zackular_AbAOMDSS_mSphere_2015. All of the FASTQ sequence data obtained in this study can be obtained from the Sequence Read Archive at NCBI under accession no. SRP056144 .
