# Supplementary figures and images for: Manipulation of the Gut Microbiota Reveals Role in Colon Tumorigenesis
Source: mSphere. 2015 Nov 4;1(1):e00001-15. doi: 10.1128/mSphere.00001-15 (PMC4863627; doi:10.1128/mSphere.00001-15)

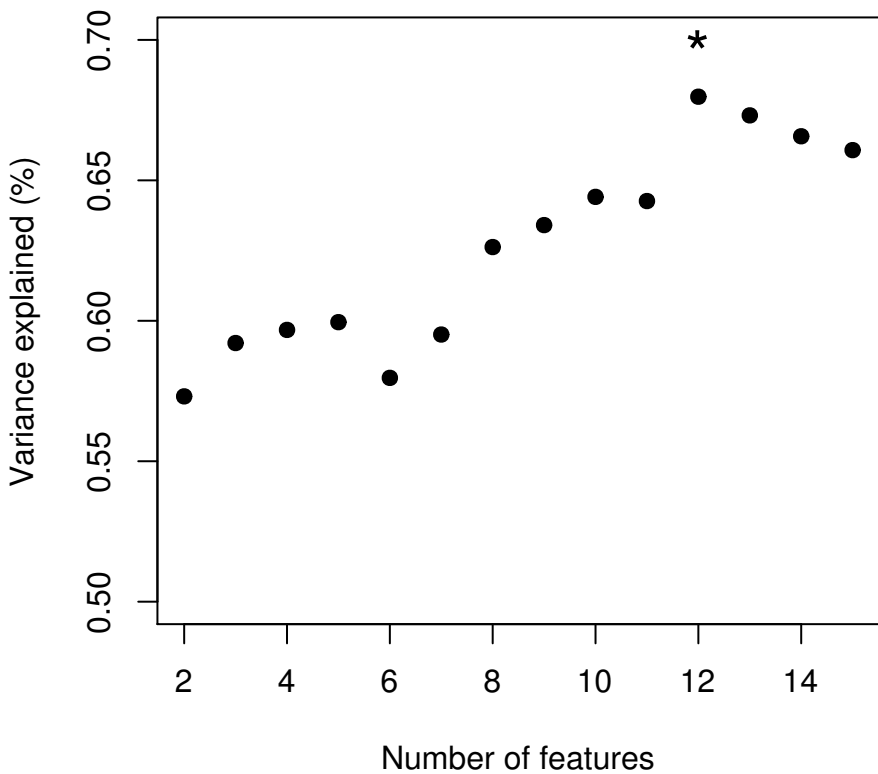

Supplement: Figure S1 [file sph001160009sf1.pdf]

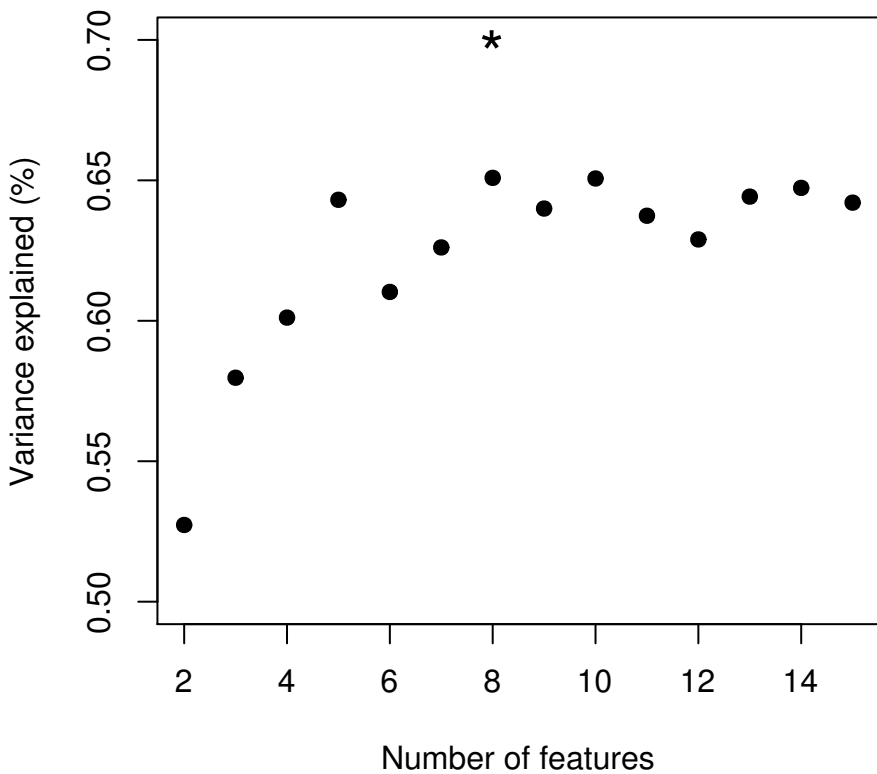

Supplement: Figure S2 [file sph001160009sf2.pdf]

Observed number of tumors

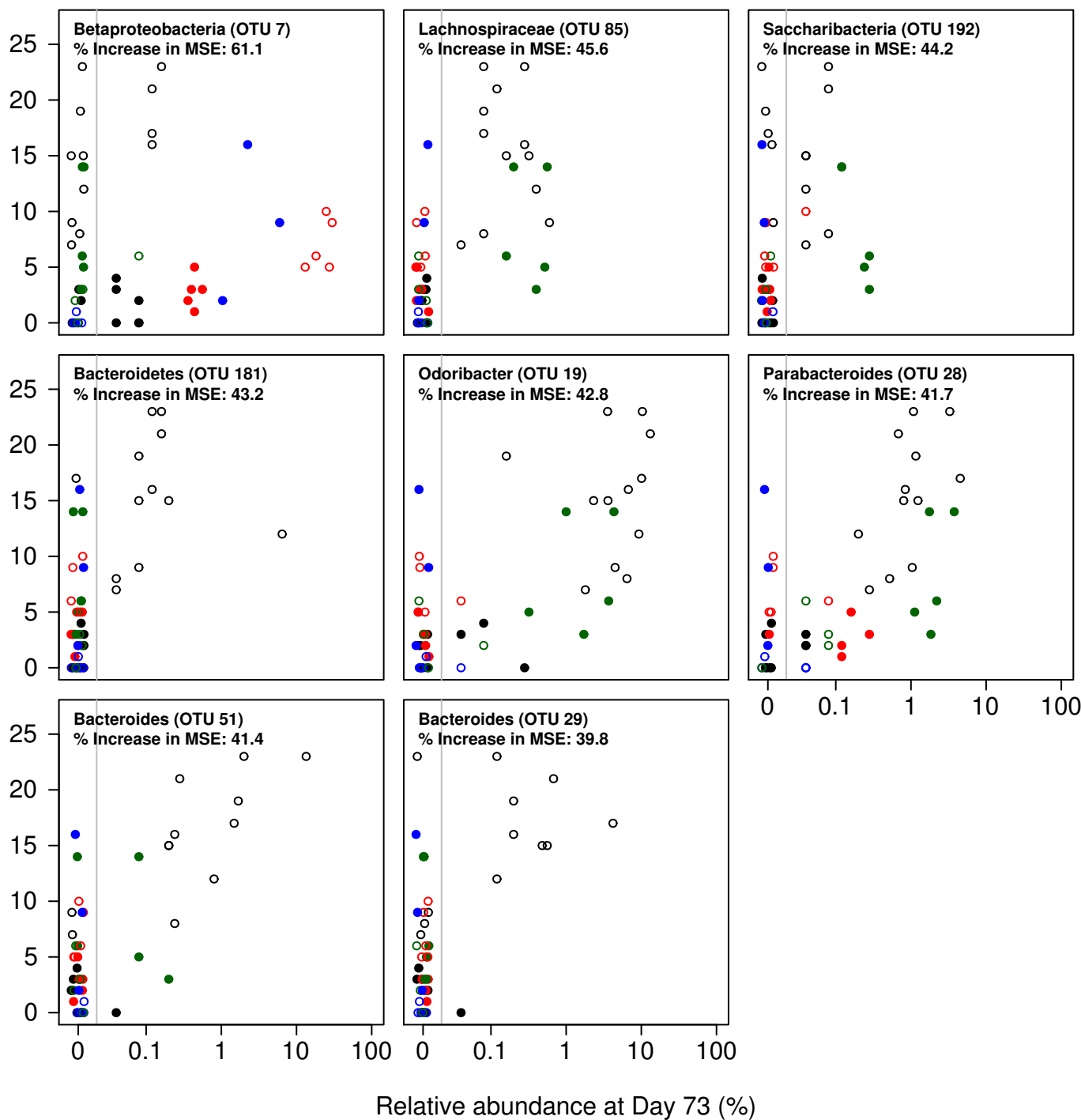

Supplement: Figure S4 [file sph001160009sf4.pdf]
