# Supplementary material for: Manipulation of the Gut Microbiota Reveals Role in Colon Tumorigenesis
Source: mSphere. 2015 Nov 4;1(1):e00001-15. doi: 10.1128/mSphere.00001-15 (PMC4863627; doi:10.1128/mSphere.00001-15)
Supplement: Figure S3 [file sph001160009sf3.pdf]

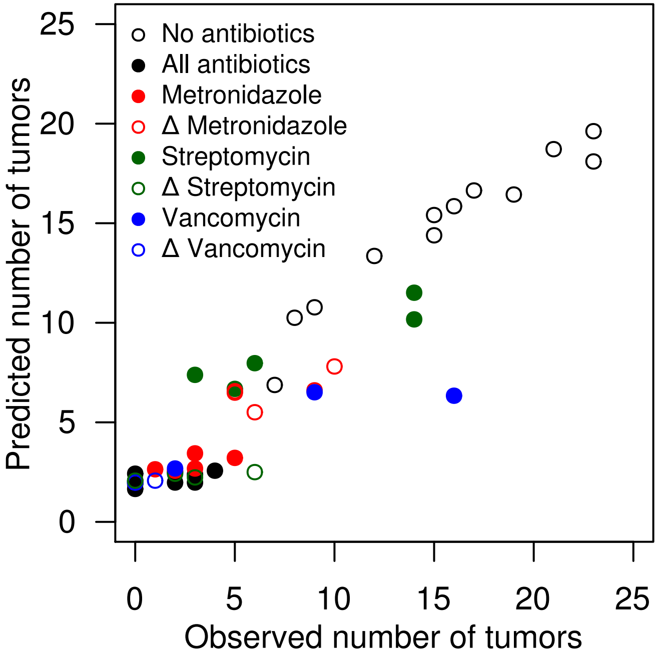
No antibiotics
All antibiotics
Metronidazole
Δ Metronidazole
Streptomycin
Δ Streptomycin
Vancomycin
Δ Vancomycin
Predicted number of tumors
Observed number of tumors
0
5
10
15
20
25
